# A cuproptosis-related lncRNA signature-based prognostic model featuring on metastasis and drug selection strategy for patients with lung adenocarcinoma

**DOI:** 10.3389/fphar.2023.1236655

**Published:** 2023-09-07

**Authors:** Mengzhe Zhang, Zengtuan Xiao, Yongjie Xie, Zekun Li, Lianmin Zhang, Zhenfa Zhang

**Affiliations:** ^1^ Department of Lung Cancer Surgery, Tianjin Lung Cancer Center, Tianjin Medical University Cancer Institute and Hospital, National Clinical Research Center for Cancer, Key Laboratory of Cancer Prevention and Therapy, Tianjin’s Clinical Research Center for Cancer, Tianjin, China; ^2^ Department of Immunology, Biochemistry and Molecular Biology, Collaborative Innovation Center of Tianjin for Medical Epigenetics, Tianjin Medical University, Tianjin, China; ^3^ Department of Pancreatic Cancer, Tianjin Medical University Cancer Institute and Hospital, National Clinical Research Center for Cancer, Key Laboratory of Cancer Prevention and Therapy, Tianjin’s Clinical Research Center for Cancer, Tianjin, China

**Keywords:** lung cancer, cuproptosis, lung adenocarcinoma, lncRNA, prognostic model, biofunction, anti-cancer drug, metastasis

## Abstract

**Introduction:** Lung adenocarcinoma is a common cause of mortality in patients with cancer. Recent studies have indicated that copper-related cell death may not occur in the same way as previously described. Long non-coding RNAs (lncRNAs) play a key role in the occurrence and development of tumors; however, the relationship between cuproptosis and lncRNAs in tumorigenesis and lung adenocarcinoma (LUAD) treatment has not been well established. Our study aimed to construct a model to analyze the prognosis of lung adenocarcinoma in patients using a carcinogenesis-related lncRNA (CR) signature.

**Methods:** The transcriptional profiles of 507 samples from The Cancer Genome Atlas were assessed. Cox regression and co-expression analyses, and the least absolute shrinkage and selection operator (LASSO) were used to filter the CR and develop the model. The expression status of the six prognostic CRs was used to classify all samples into high- and low-risk groups. The overall disease-free survival rate was compared between the two groups. The Gene Ontology and the Kyoto Encyclopedia of Genes and Genomes were used to identify the pathways and mechanisms involved in this model. Subsequently, immunotherapy response, sensitivity, and correlation analyses for several anti-tumor medications were performed. *In vitro* experiments, including qPCR, were conducted in nine lung adenocarcinoma cell lines and 16 pairs of lung adenocarcinoma and para-carcinoma tissues.

**Results:** After confirmation using the ROC curve, patients in the low-risk category benefited from both overall and disease-free survival. Gene Ontology analysis highlighted cell movement in the model. In the *in vitro* experiments, qPCR results showed the expression levels of six CRs in 16 pairs of carcinoma and para-carcinoma tissues, which were in accordance with the results of the model. AL138778.1 is a protective factor that can weaken the invasion and migration of A549 cells, and AL360270.1 is a hazardous factor that promotes the invasion and migration of A549 cells. According to this model, targeted treatments such as axitinib, gefitinib, linsitinib, pazopanib, and sorafenib may be more appropriate for low-risk patients.

**Conclusion:** Six CR profiles (AL360270.1, AL138778.1, CDKN2A-DT, AP003778.1, LINC02718, and AC034102.8) with predictive values may be used to evaluate the prognosis of patients with lung adenocarcinoma undergoing therapy.

## 1 Introduction

Lung adenocarcinoma (LUAD) is a malignancy of the respiratory system and is highly prevalent worldwide (Sequist et al., 2013). To better understand and develop more therapeutic mechanisms for patients with LUAD, researchers have established risk models to classify patients and implement proper treatment plans and drug selection strategies (Gautschi et al., 2020). Copper (Cu) elements are involved in various biological functions (van den Berghe and Klomp, 2009). Recent studies have revealed that Cu concentrations are strongly enriched in tumor tissues when compared to that of normal tissues (Tsang et al., 2020). Negative effects, such as cancer pathogenesis, have been associated with high concentrations of Cu that exceed the threshold (Ishida et al., 2013). Dysregulation of Cu has been linked to cancer development (Sciegienka et al., 2017). Cu-based promoters and antagonists have therefore been used as anti-tumor agents (Brady et al., 2017). It has been noted that Cu molecules bind compactly to the tricarboxylic acid cycle, resulting in the accumulation of toxic proteins and cell death (Solmonson et al., 2022). A previous study also demonstrated that cuproptosis-related genes (CG) could induce many cell-related pathways, including apoptosis, autophagy and anti-angiogenesis (Xie et al., 2023). DNA is extensively transcribed and produces many long non-coding RNAs (lncRNAs). These lncRNAs are more than 200 nucleotides in length and are not translated into functional proteins. LncRNAs regulate the biological behavior of cancer cells and are associated with the pathogenesis and progression of various cancers (Zhang et al., 2022). Several reports have demonstrated that lncRNAs are associated with cuproptosis. The genes and lncRNAs involved in this process have been identified, and this has led to further exploration into their roles in neoplasm development and invasion via transcriptional modifications (Loewen et al., 2014). Emerging evidence suggests that the dysregulation of lncRNAs in LUAD is widely involved in tumor cell proliferation, invasion, and metastasis, as well as shaping the TME (Cobine and Brady, 2022). Studying cuproptosis-related lncRNAs may provide further insight into the role of this pathway in cancer including the PI3K/AKT, NF-κB, p53, and Notch pathways (Ritchie et al., 2015). Additionally, lncRNAs are largely associated with drug resistance in tumors (Dong et al., 2019). Whether lncRNAs are related to tumor invasion and migration remains unclear, and the pathways should be explored. In this study, we analyzed the cuproptosis-related genes (CG) in LUAD. We also generated a model of carcinogenesis-related lncRNAs (CR) to predict the prognosis of LUAD.

## 2 Materials and methods

### 2.1 Data collection and identification of CRs

We extracted data from The Cancer Genome Atlas (TCGA) and GEO datasets using Perl (version 5.30.0–64 bit). The software R (version 4.0.1) and GraphPad Prism (version 8.0.2) were used for data analysis.

### 2.2 Creation and validation of cuproptosis-related lncRNAs

The lncRNAs in the CGs were screened using the Pearson correlation method. The candidate CR (*p* < 0.05) was selected for further analysis. Univariate Cox regression analysis was conducted to identify lncRNAs that were linked to the prognosis. These lncRNAs were mapped by the “limma,” for the classifying the lncRNAs; “ggplot2,” for drawing the graph; “heatmap,” for classifying the CRs; “survminer,” for survival analysis and visualization; “timeROC,” for calculating the cut-off point and the area; and “caret” packages for model prediction and testing. By applying the least absolute shrinkage and selection operator (LASSO) analysis to these lncRNAs, we identified the most suitable group of prognostic lncRNAs. After multi-Cox regression analysis, we established a 6-lncRNAs-risk model as follows (Eq. (1)):

$$Risk\;score=\sum i=lnCoef\left(i\right)\times Expr\left(i\right)$$ (1)

where Coef (i) refers to each lncRNA’s regression coefficient in the multiple Cox regression analysis, and Expr (i) refers to each lncRNA’s normalized expression level. We categorized the above-mentioned lncRNAs into a low-risk group (LRG) with a hazard ratio (HR) < 1 and a high-risk group (HRG) with an HR > 1.

### 2.3 Construction of nomogram and calibration

The 1-, 3-, and 5-year overall survival (OS) in all samples was presented by figures drawn using the “survival,” for analyzing the survival of the cases, “regplot,” for fitting the regression model and “rms,” for significance analysis of output variables. We applied risk scores to different clinicopathological factors, and a calibration curve was drawn according to the Hosmer-Lemeshow method.

### 2.4 Principal component, Gene Ontology, and KEGG analyses

To observe the different spatial distributions in the LRG and HRG, we used principal component analysis (PCA) to investigate the expression status of CRs in patients with LUAD. Firstly, we applied the Gene Ontology (GO) analysis (GO; http://www.geneontology.org/) by using “clusterProfiler” for gene enrichment; “colorspace” “stringi”, “ggplot2”, “CRclize” and “RcolorBrewer” for drawing the circle-map. Finally, we showed the difference of cellular components, molecular functions, and molecular biological processes. The Kyoto Encyclopedia of Genes and Genomes (KEGG; http://www.genome.jp/kegg/) pathways that were differentially expressed were analyzed by using “clusterProfiler”, “enrichplot”, “ggplot2”, “dplyr” and “ComplexHeatmap”. We considered the enriched biological functions, processes, and pathways significant when *p* < 0.05.

### 2.5 Tumor-immune-related function analysis

We determined the immune infiltration profile by using the “limma,” for processing the gene expression matrix and “BiocManager” for visualization of data.

### 2.6 Tumor mutational burden and therapeutic drug correlation sensitivity analysis

Pearl was used to download the mutation data. “Map tools” was used to capture the mutational characteristics of the tumor mutational burden (TMB) and survival in LRG and HRG. All Tumor Immune Dysfunction and Exclusion (TIDE) files were obtained from http://tide.dfci.harvard.edu. We utilized “pRRophetic” for predicting the correlation between the half-limiting dose (IC50 values) and the risk scores. Finally, the sensitivity of the suitable treatment drugs was determined.

### 2.7 Validation of CRs under *in vitro* conditions

Based on the transcriptional sequences of the six lncRNAs, six qPCR primers were designed for each. All the sequences for these CRs have been explained below (5′-3′):

AL360270.1 F: CAG​TCA​TAC​CAC​CCT​GAA​CAC.

R: GGA​TTA​ACC​AGG​CCC​AAC​C.

AL138778.1 F: AGT​CTG​CAG​GAG​AAA​TGA​CTG​G.

R: AAA​AGT​GCC​TTG​GCA​AGC​AG.

CDKN2A-DT F: AGC​GTG​GAC​AGG​AGC​ATC​TC.

R: GGC​TGT​GAG​GTT​GCG​AAT​GAC.

AP003778.1 F: TAG​GTT​ATC​TGG​CAG​CAA​CTT​CAC.

R: GCA​CTT​ACT​CCA​TTC​ACG​CAT​TC.

LINC02718 F: AGC​CGA​CTG​TGG​GAC​CTT​G.

R: GCA​TCT​GCT​CCT​TCC​ATC​TTC​TAC.

AC034102.8 F: GTG​GTG​GTG​TGG​CTC​ATT​GTG.

R: TGG​CTC​CTG​TGG​CTG​TAT​CTG.

GAPDH F: GGT​GGT​CTC​CTC​TGA​CTT​CAA​CA.

R: GTT​GCT​GTA​GCC​AAA​TTC​GTT​GT.

To validate the above CRs, we used 16 pairs of adenocarcinomas and their para-carcinoma tissues to determine the different expression levels of lncRNAs in this model.

For further confirmation, we selected nine types of adenocarcinomas in cell lines, including BEAS2B, A549, PC9, HCC827, H1299, H1650, H1975, H358, and H441. We selected the lowest and highest lncRNAs in our model to compare their relative expression levels in the 9 cell lines.

We isolated RNA from 16 pairs of tumors and para-carcinoma tissues. Information on the samples is shown in Supplementary Table S1. cDNA was prepared for CRs and GAPDH. Real-time qPCR was performed to determine the expression levels of the six lncRNAs in all samples. All staining was based on the SYBR Green Master (ROX, Roche; United States).

### 2.8 Cell culture and reagents

Human BEAS-2B (BEAS-2B was used for comparison), A549, PC9, HCC827, H1299, H1650, H1975, H358, and H441 cell lines were obtained from The American Type Culture Collection. Cells were maintained in Roswell Park Memorial Institute (RPMI) 1,640 medium (Gibco, United States) containing 10% fetal bovine serum (FBS) (Sigma, United States), streptomycin (50 g/mL) (Sigma, United States), and penicillin (100 U/mL) (Hyclone, United States). Cells were cultured in a incubator with 37°C humidified and 5% CO_2_ incubator (SANYO, Japan).

### 2.9 Plasmid construction and lentivirus packaging

The AL138778.1 and AL360270.1 transcripts were cloned into the pCDH lentiviral vector, and lentiviral shRNAs targeting AL138778.1 and shRNA targeting AL360270.1 were obtained from Genechem (Shanghai, China). The lentiviral vectors were used according to the manufacturer’s instructions.

### 2.10 RT-PCR analysis

RNAs were extracted from cells using TRIzol reagent (Invitrogen). After the synthesis of cDNA, RT-PCR was repeated three times. In order to determine whether targeted drugs could influence the expression of the six CRs, we performed axitinib (10 nM) incubations with the wild-type A549 cell line for 6 h in a 24-well plate and then performed qPCR to determine the change in the six lncRNAs (Wang et al., 2019).

### 2.11 Scratch wound and transwell assay-the wound healing assay

We constructed the AL138778.1 knock-down and AL138778.1 control vector, and the AL360270.1 knock-down and AL138778.1 control vector in the A549 cell line. We then inoculated the cells (1 × 10^5^/well) into a 24-well culture plate and cultivated them in an environment of 37°C, 5% CO_2_ for 24 h. The culture solution was discarded, and a 10 μL pipette tip was used to scratch the inoculated cells. The cells were then gently washed twice with PBS, and 1 mL RPMI 1640 medium was added. A photograph of each scratch was taken at 0 and 24 h. Each experiment was conducted in 3-line parts and repeated five times. We measured and calculated the migration distance from the original site to the wounded area over 24 h. We placed the cells (5 × 10^4^ cells/well) in a Matrigel plate well containing serum-free RPMI 1640 medium. The lower chamber was then filled with 500 µL of complete medium (RPMI 1640 with 10% FBS). The cells that did not pass the well were lightly cleaned with a cotton swab after incubation at 37°C for 24 h. Glutaraldehyde (5%) was then added in the lower chamber, and the cells were allowed to fix for 10 min. Crystal violet (1%) in 2% ethanol was used to stain the cells at room temperature (approximate temperature range from 15°C to 20°C) for 20 min. We used inverted microscope (OLYMPAS, Japan) to photograph five different sites and counted the numbers in a 10× view average for comparison.

### 2.12 Western blot analysis

We chose the following proteins that are closely related to migration in lung cancer (Bremnes et al., 2002): N-cadherin, E-cadherin, Vimentin, Snail, and Sox2. All data were normalized to those of GAPDH. We washed the cells three times with PBS, and then lysed them. After SDS-PAGE, the proteins were transferred onto polyvinylidene difluoride (PVDF) membranes by Western blotting. Skimmed milk (4%) was used to block the blots for 1 hour, and the primary monoclonal antibodies against the proteins (N-cadherin, E-cadherin, Vimentin, Snail, Sox2, and GAPDH) were added and incubated at 4°C overnight. Membranes were incubated with secondary antibodies (1:1000 dilution) for 1 hour at room temperature. The membrane strips were then exposed to enhanced chemiluminescence and a fixer (1:1). The details of all the antibodies are shown in Supplementary Table S1.

## 3 Results

### 3.1 lncRNAs from Co-expressional cuproptosis-related genes

**FIGURE 1:**
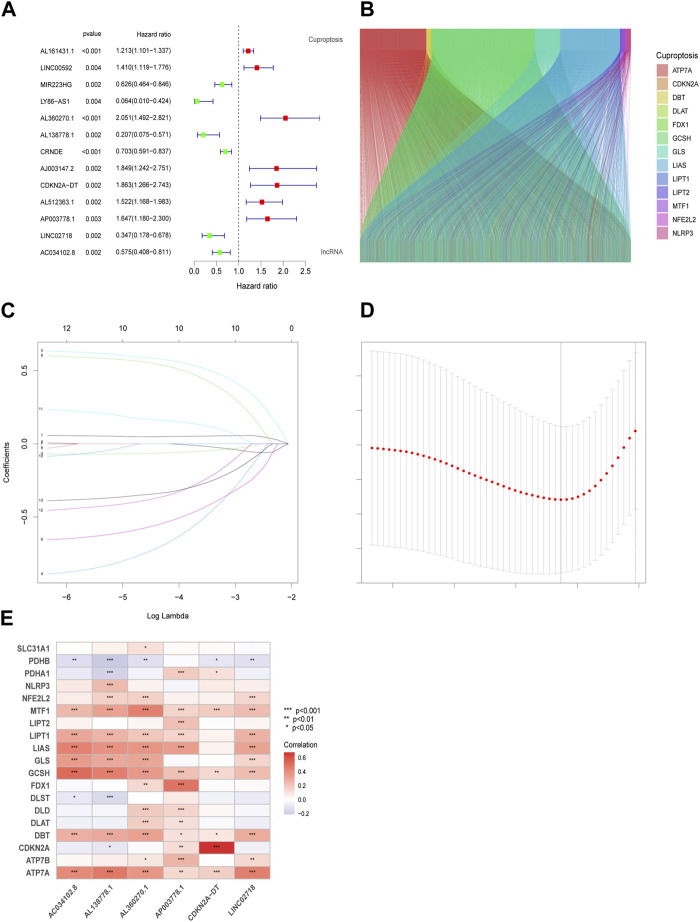
Prognostic features in the identification of Cuproptosis-associated lncRNA (CR). Thirteen eligible CRs with a *p* < 0.05 were chosen by uniforest plot **(A)**. The Sankey diagram revealed the corelation in cuproptosis genes and CRs **(B)**. Variable selection based on 10-fold cross-validation with the least absolute shrinkage and selection operator (LASSO) algorithm **(C)**. Coefficient LASSO patterns for CRs **(D)**. Heatmap of the corelation between CRs and CGs in risk models **(E)**.

After risk score analysis, all LUAD cases in the test (n = 339) and training groups (n = 168) were separated into LRG and HRG. The basic clinical factors are presented in Table 1. A total of 16,876 lncRNAs from 19 cuproptosis-related genes (CG) were selected using Pearson correlation analysis. Univariate Cox regression analysis was used to identify the lncRNAs related to cuproptosis. Finally, 13 eligible CRs were selected (*p* < 0.05; Figure 1A). The co-expression network of LUAD is shown in Figure 1B. An expression heatmap of the six lncRNAs in the LRG and HRG is shown in Figure 1E.

**TABLE 1:** The basic characteristic of the samples (age, gender, and TNMstage) in our baseline analysis. Data were presented as numbers (Percentage %).

Characteristics	Type	Total (n = 507)	Test group (n = 168)	Training group (n = 339)	*p*-Value
Age	≤65	239 (47.14%)	72 (42.86%)	167 (49.26%)	0.1378
	>65	258 (50.89%)	95 (56.55%)	163 (48.08%)	
	Unknown	10 (1.97%)	1 (0.6%)	9 (2.65%)	
Gender	Female	272 (53.65%)	89 (52.98%)	183 (53.98%)	0.9051
	Male	235 (46.35%)	79 (47.02%)	156 (46.02%)	
TNM Stage	I	272 (53.65%)	88 (52.38%)	184 (54.28%)	0.5459
	II	120 (23.67%)	38 (22.62%)	82 (24.19%)	
	III	81 (15.98%)	32 (19.05%)	49 (14.45%)	
	IV	26 (5.13%)	7 (4.17%)	19 (5.6%)	
	Missing/unknown	8 (1.58%)	3 (1.79%)	5 (1.47%)	
T stage	T1	169 (33.33%)	57 (33.93%)	112 (33.04%)	0.9926
	T2	271 (53.45%)	88 (52.38%)	183 (53.98%)	
	T3	45 (8.88%)	15 (8.93%)	30 (8.85%)	
	T4	19 (3.75%)	6 (3.57%)	13 (3.83%)	
	Unknown	3 (0.59%)	2 (1.19%)	1 (0.29%)	
N stage	N0	327 (64.5%)	104 (61.9%)	223 (65.78%)	0.6264
	N1	95 (18.74%)	33 (19.64%)	62 (18.29%)	
	N2	71 (14%)	26 (15.48%)	45 (13.27%)	
	N3	2 (0.39%)	0 (0%)	2 (0.59%)	
	Unknown	12 (2.37%)	5 (2.98%)	7 (2.06%)	
M stage	M0	338 (66.67%)	114 (67.86%)	224 (66.08%)	0.7141
	M1	25 (4.93%)	7 (4.17%)	18 (5.31%)	
	Unknown	144 (28.4%)	47 (27.98%)	97 (28.61%)	

### 3.2 Construction of the predictive signature

We used the “caret,” “glmnet” to perform a LASSO analysis in the training group to choose lncRNAs that possessed the best prognostic value (Figures 1C,D). Using the results from Eq. (1), the value of each lncRNA was calculated (Eq. (2)):

$$“risk\;scores”=AL360270.1\times \left(0.672984389012466\right)+AL138778.1\times \left(-0.998712907510809\right){+}^{"}CDKN2A-DT^{"}\times \left(0.603568904030502\right)+AP003778.1\times \left(0.250123284609668\right)+LINC02718\times \left(-0.528689990460446\right)+AC034102.8\times \left(-0.472815056249193\right)$$ (2)

**FIGURE 2:**
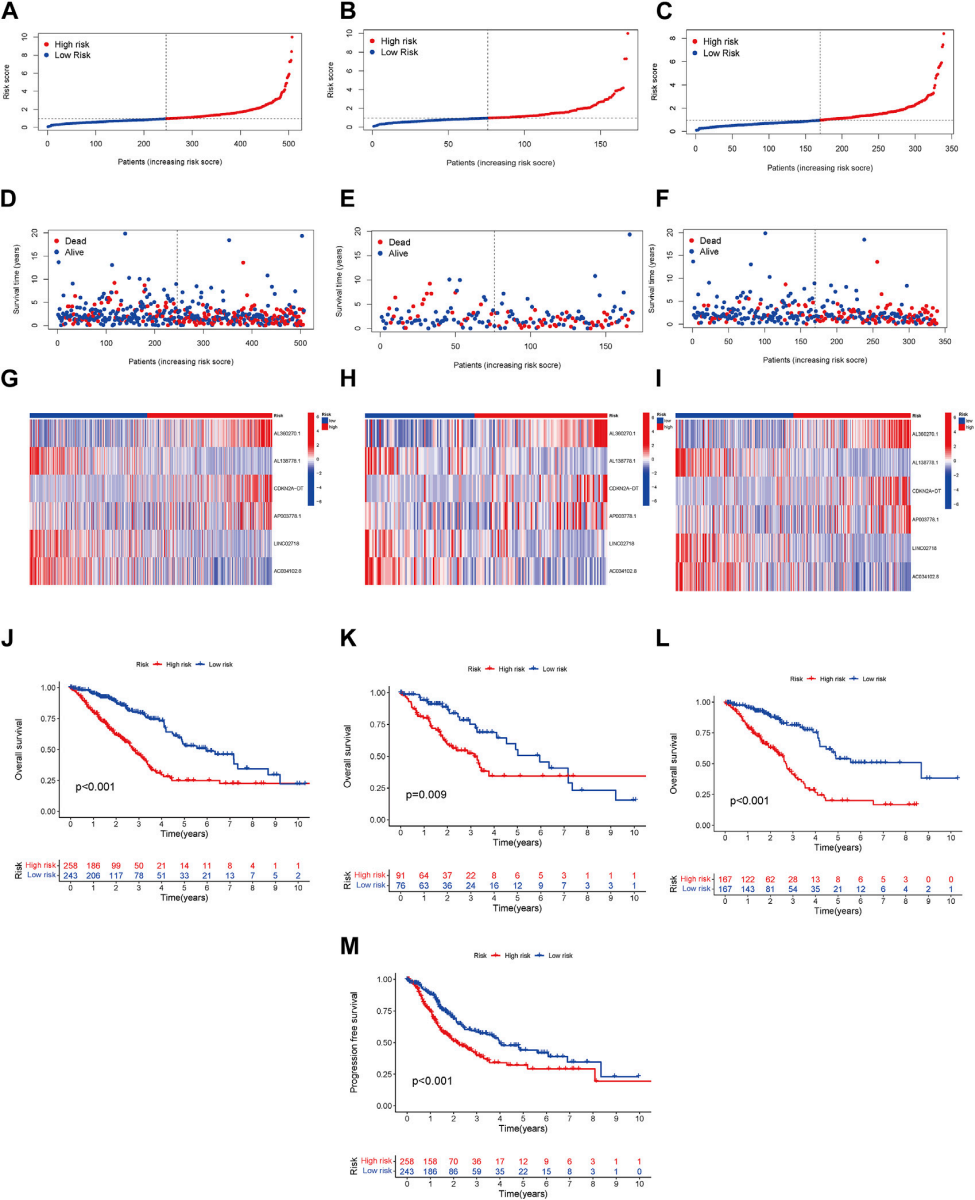
Prognosis model linked with risk in LRG and HRG. The overall survival (OS) risk scores (including all samples, test, and training groups) **(A–C)**. Survival characteristics in three cohorts (including all samples, test, and training groups) **(D–F)**. The heatmaps of 6 lncRNA expressions, (including all samples, test, and training groups) **(G–I)**. The LUAD patients’ OS in three cohorts (including all samples, test, and training groups) **(J–L)**. The LUAD patients’ PFS in all samples **(M)**.

We conducted a patient prognostic analysis for the LRG and HRG. In the three cohorts (all samples, test, and training groups), the risk scores were significantly higher in the HRG (Figures 2A–C). The survival time in the LRG was longer than that of the HRG in all three cohorts (Figures 2D,E). The heatmap shows the expression status and correlation of the six lncRNAs in the three cohorts. Patients in the LRG showed a negative relationship with the risk factors AL360270.1, CDKN2A-DT, and AP003778.1. In contrast, patients in the HRG showed a positive relationship with these factors (Figures 2G,H). Patients in the LRG had a better OS than those in the HRG in all groups (Figures 2J–L). Patients in the LRG also benefited from improved progression-free survival (PFS) compared to those in the HRG (Figure 2M).

### 3.3 The risk score presented an indicative value in this signature

**FIGURE 3:**
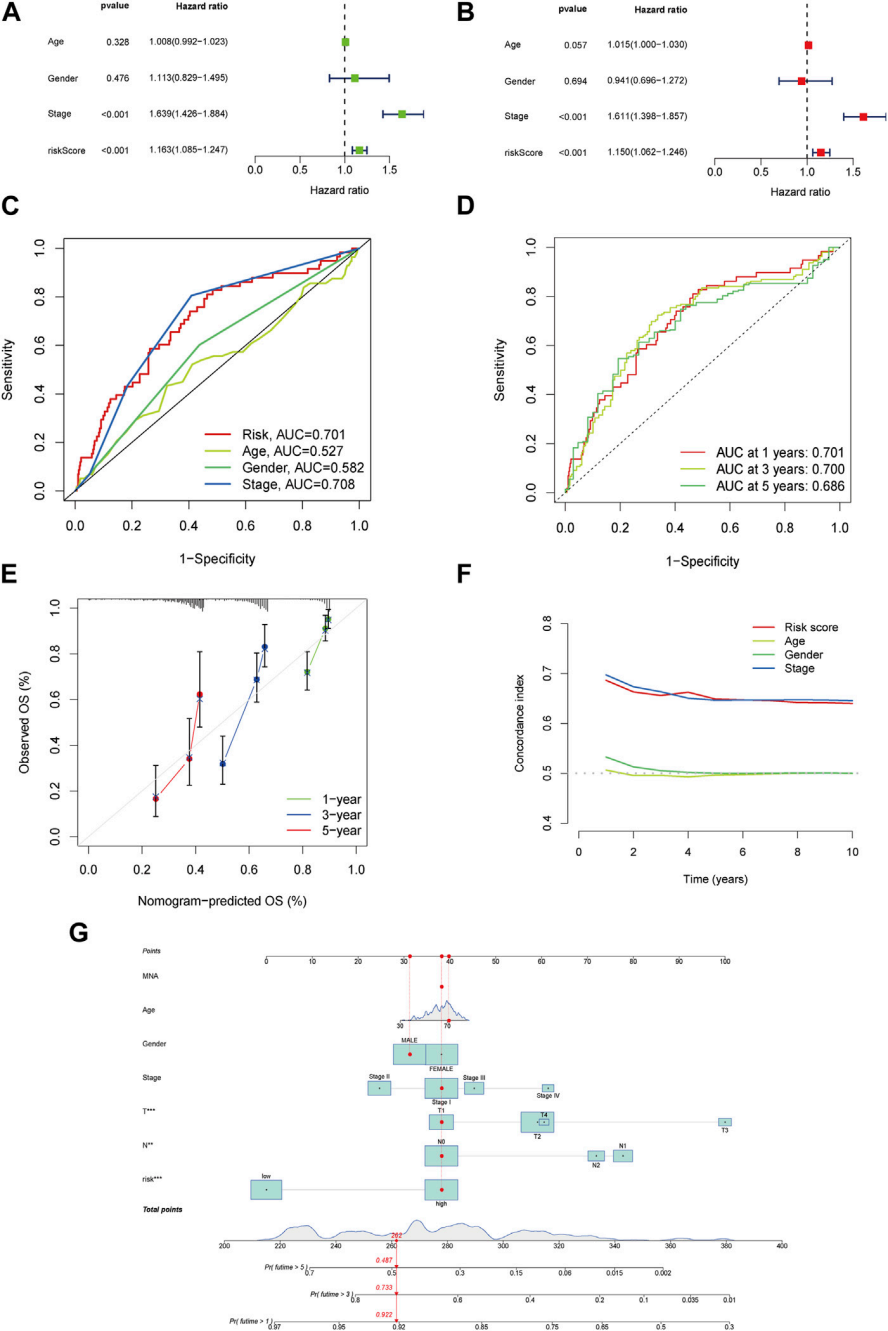
The risk model’s accuracy and the nomogram’s validation. Univariate analysis for the clinical features compared with the risk score **(A)**. Multivariate analysis for risk scores and clinical characteristics **(B)**. ROC curves summarized the risk characteristic for 1 year, 3 years, and 5 years **(C)**. ROC curves’ characteristics in age, sex, and stage in the clinical model **(D)**. Calibration curves test the accuracy in this model at 1 year, 3 years, and 5 years **(E)**. Zero-10 years’ C-index curve **(F)**. A nomogram shows 1 year, 3 years, and 5 years’ OS in LUAD patients based on risk scores along with clinicopathological characteristics **(G)**.

Based on the Cox regression analysis, the risk score was more efficient than other characteristics ([analysis of univariation: HR = 1.163, 95% CI = 1.085–1.247, *p* < 0.001] and [analysis of multivariation: HR = 1.150, 95% CI = 1.062–1.246, *p* < 0.001]), as shown in Figures 3A,B. The ROC curve also highlighted the sensitivity and specificity of the risk score, which was more efficient than those of age and sex (Figure 3C; risk score: AUC = 0.701). Similarly, the model presented a predictive value with high sensitivity (Figure 3D; AUC of 1 year = 0.701, AUC of 3 years = 0.700, and AUC of 5 years = 0.686).

### 3.4 Validation and accuracy of the risk model and nomogram

The calibration curves agreed well with the predictors and nomogram (Figure 3E). In the risk model, the C-index was higher than age and sex, especially in the 8th –10th years (Figure 3F). All samples at one, three, and 5 years were included in the nomogram and combined with their characteristics and risk scores (Figure 3G). In addition, the ROC and nomogram analyses demonstrated the accuracy of the signature.

### 3.5 Survival curves based on TNM stage

**FIGURE 4:**
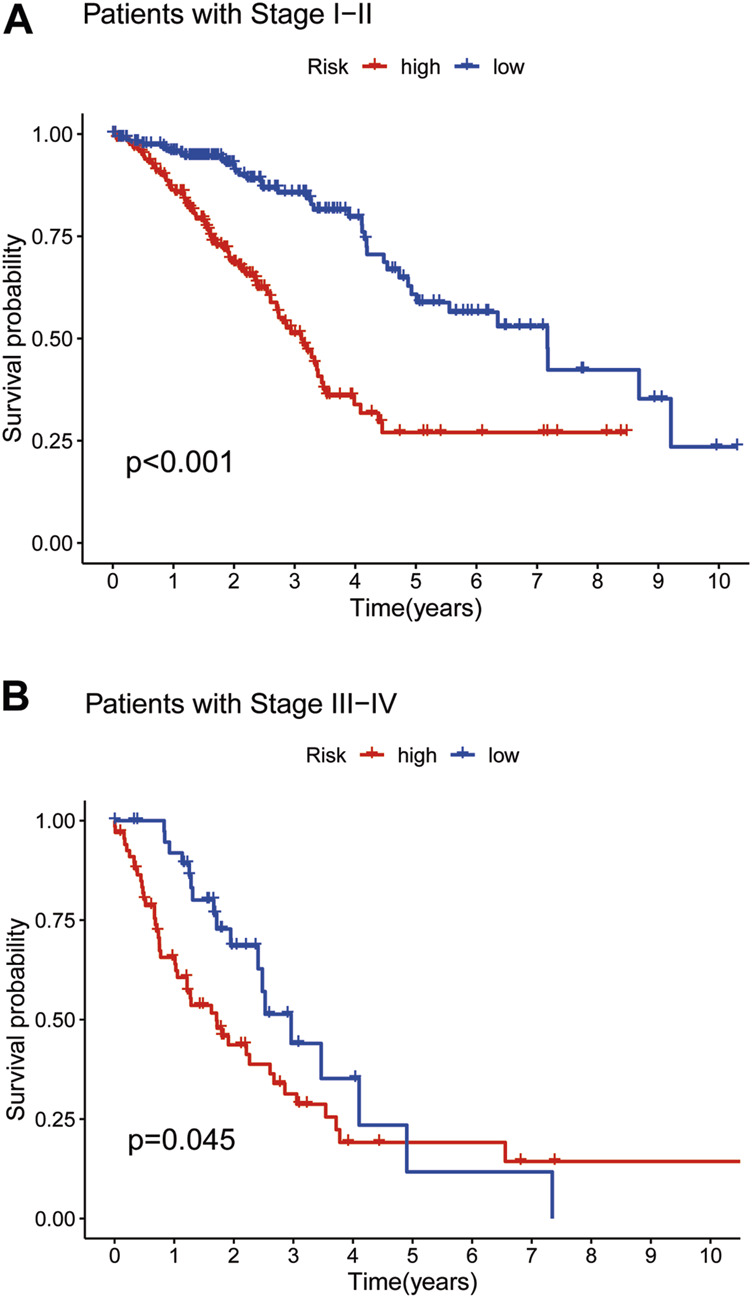
The Kaplan–Meier survival curves in low- and high-risk populations by TNMstage. Analysis of OS of patients with stage I−II in two groups **(A)**. Analysis of OS of patients with stage III−IV in two groups **(B)**.

To further validate survival, we divided patient prognosis into stages (stages I−II and stages III−IV) for the survival probability analysis. The LRG benefited more than the HRG (Figures 4A,B; *p* < 0.001, *p* = 0.045, respectively).

### 3.6 Establishment and presentation of the principal component analysis

**FIGURE 5:**
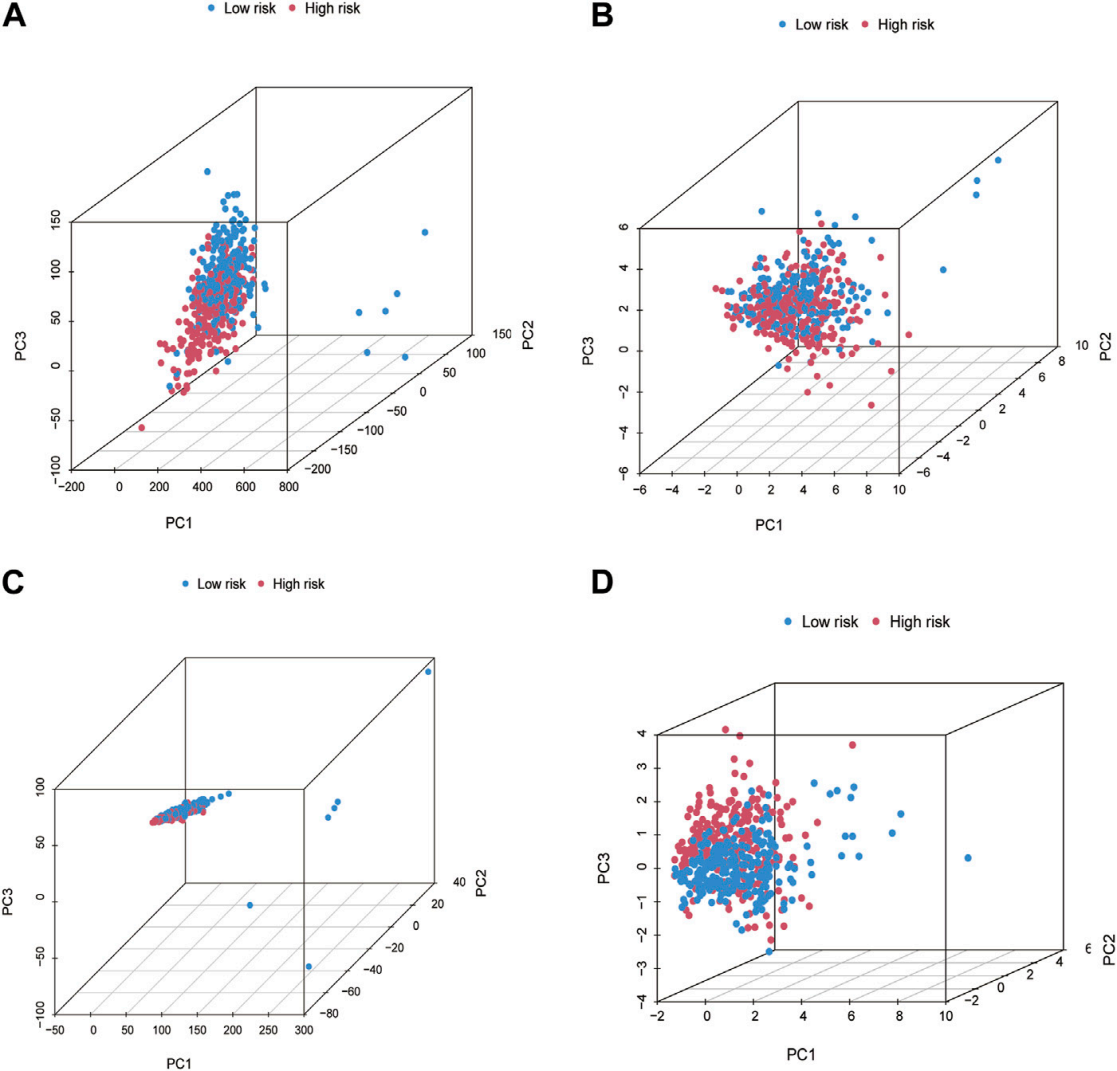
The principal component analysis (PCA) for patients in LRG and HRG. PCA in All genes **(A)**. Cuproptosis genes **(B)**. Cuproptosis-related lncRNAs **(C)**. Risk lncRNAs alone **(D)**.

We used PCA to determine all gene expression profiles, CR and cuproptosis genes, and risk model lncRNAs in both groups. The outcomes indicated that all six CRs had the ability to differentiate between the LRG and HRG. The connection distance of each sample is large, and the sample composition is different (Figures 5A–D).

### 3.7 Analysis of biological pathways

**FIGURE 6:**
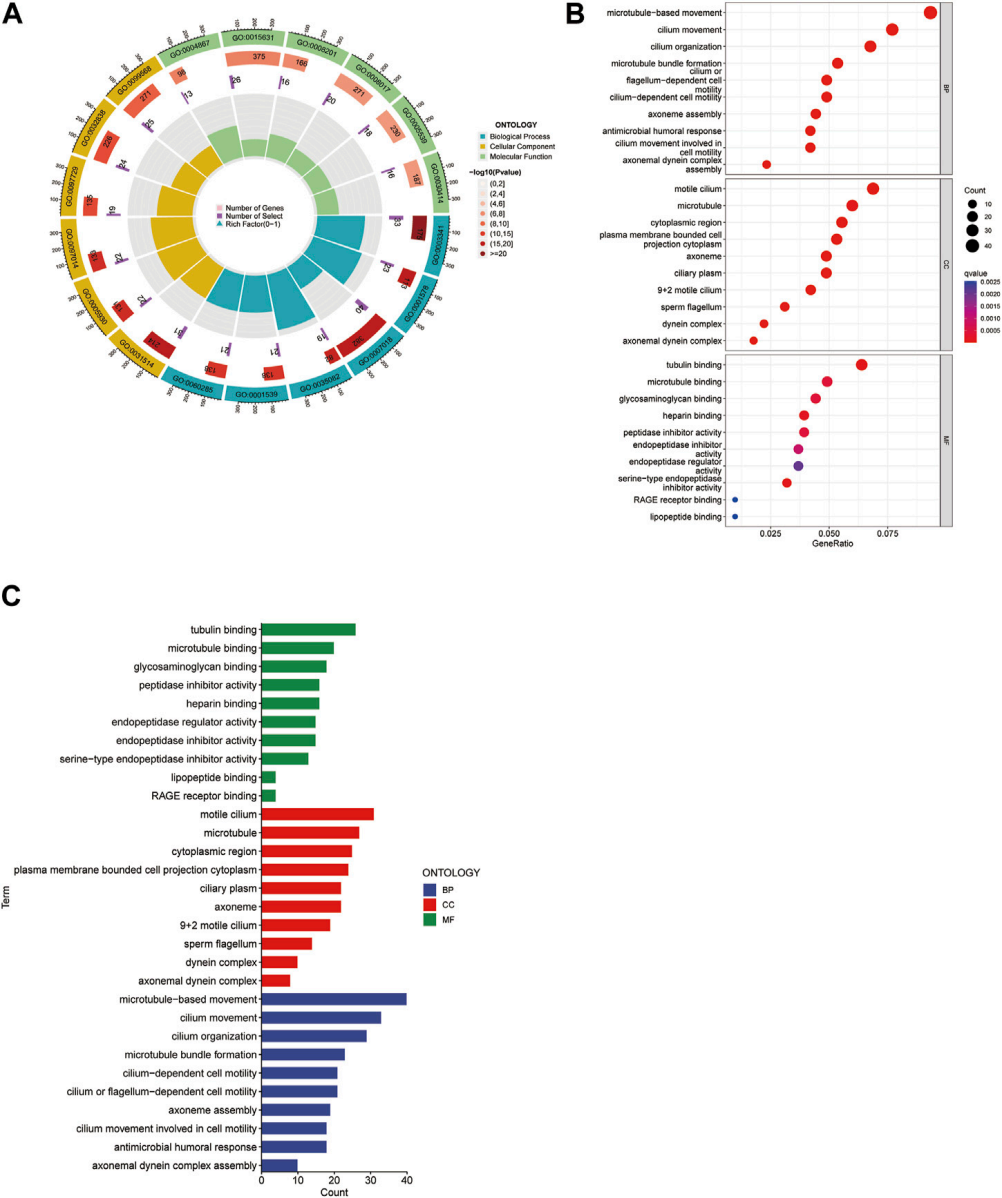
Gene Ontology (GO) analysis. The circle map presents the overview of the whole process **(A)**. The bubble map showed both the component and the **q** value **(B)**. It reveals predominant cellular components, molecular biological processes, and molecular functions **(C)**.

**FIGURE 7:**
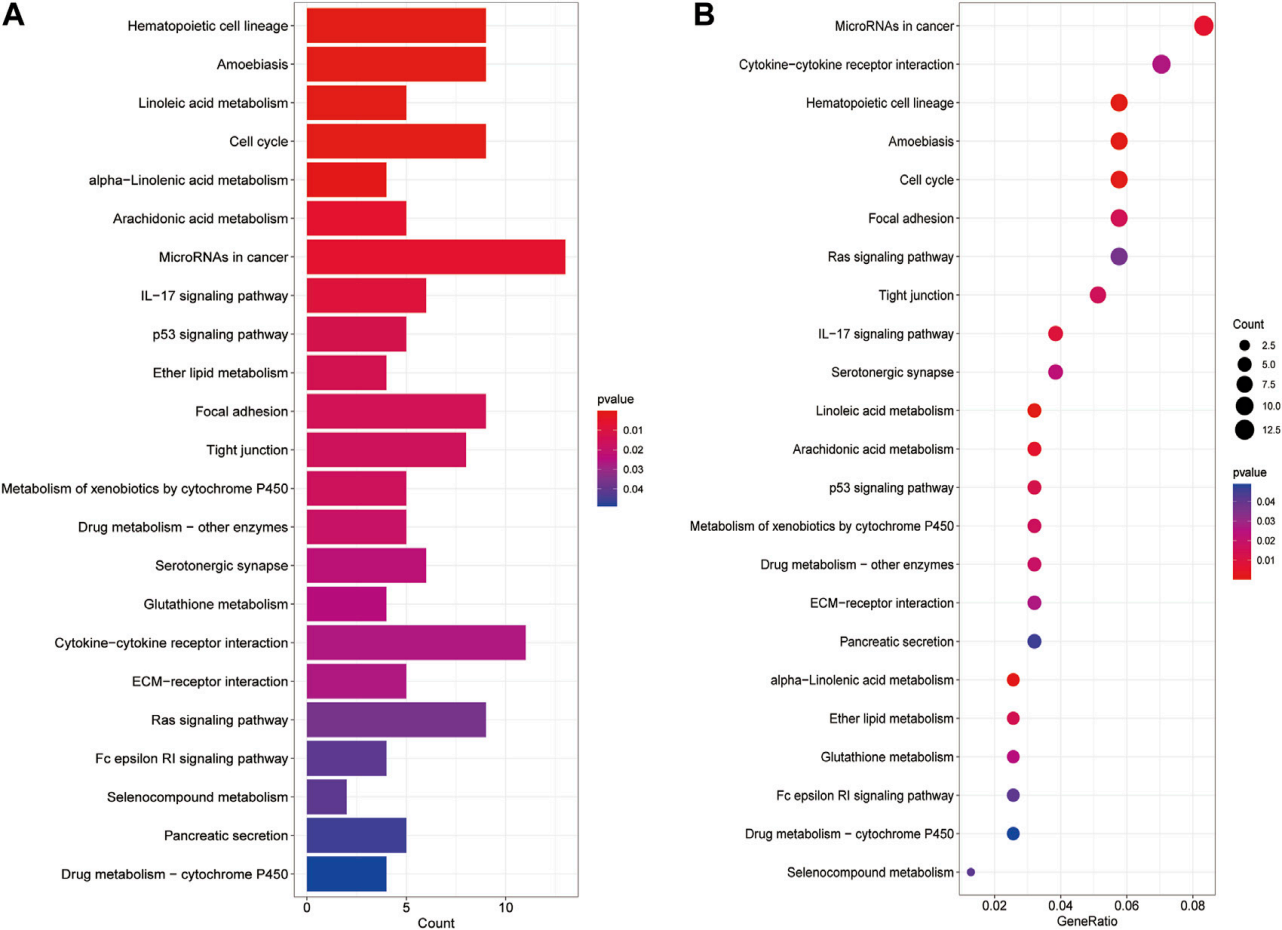
KEGG analysis shows significantly enriched KEGG pathways **(A, B)**.

We analyzed biological pathways using the GO and KEGG databases to demonstrate the different functions of cuproptosis-related genes (CGs). In GO analysis, the biological procedures, and functions of CGs mainly included tubulin binding, motile cilia in Molecular Function (MF) microtubules in Cellular Component (CC), microtubule-based movement, and cilium movement in Biological Process (BP) (Figures 6A–C). In the KEGG pathway enrichment analysis, the genes were predominantly involved in lipopeptide binding, RAGE receptor binding, endopeptidase regulatory activity, and endopeptidase inhibitors. In addition, arachidonic acid metabolism, linoleic acid metabolism, the p53 signaling pathway, and the metabolism of xenobiotics by cytochrome P450 also showed strong relevance to the model (Figures 7A,B).

### 3.8 Analysis of immune-related function, genetic alterations, TMB, TIDE, and sensitivity of the therapeutic drug

**FIGURE 8:**
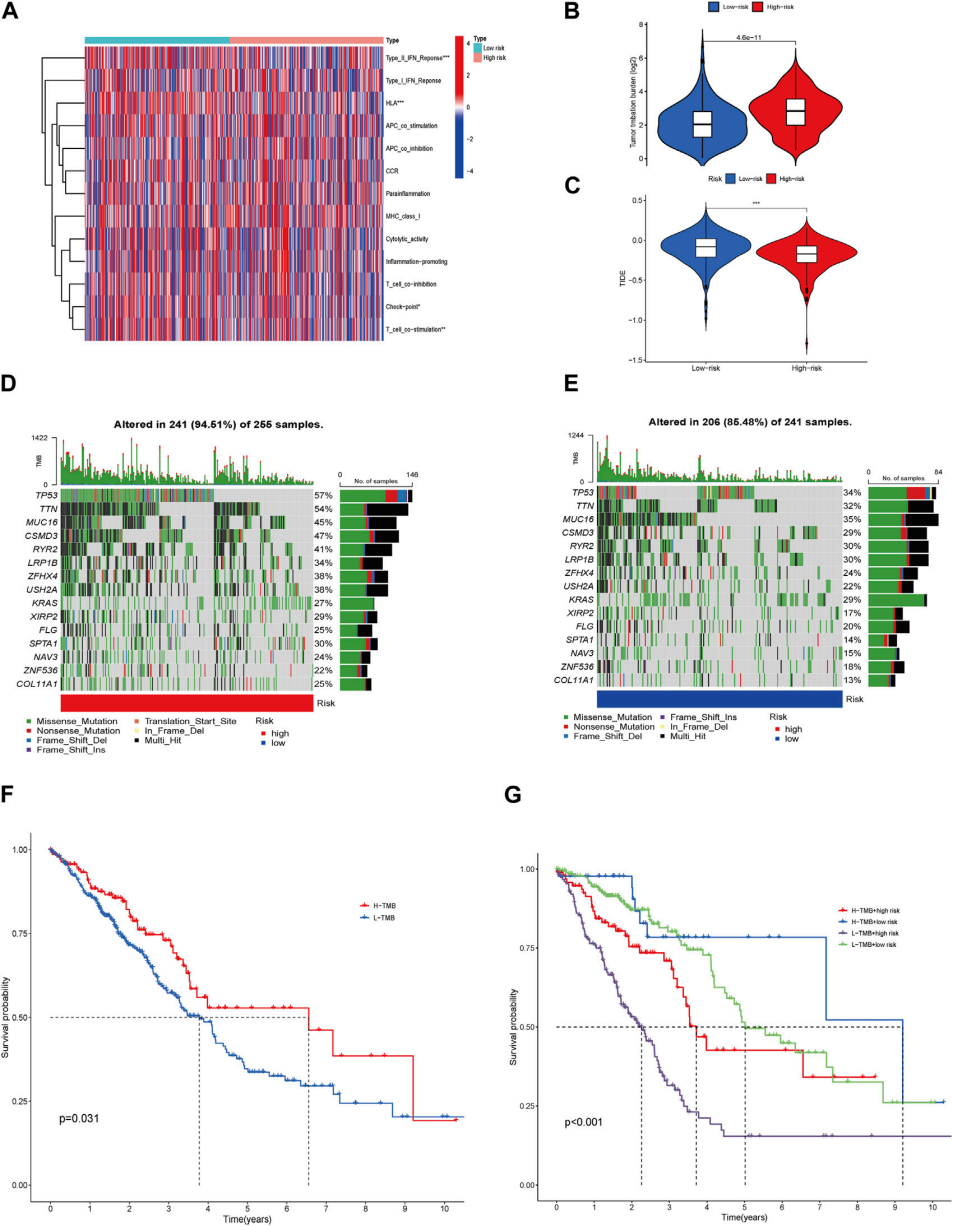
Immune-related function, Genetic alterations, TMB, TIDE, and Therapeutic Drug Sensitivity. Heatmap for various immune-related cells in LRG and HRG **(A)**. TMB in the LRG and HRG **(B)**. TIDE in the two groups **(C)**. Waterfall plots described the somatic mutation features in HRG **(D)**. Waterfall plots described the somatic mutation features in LRG **(E)**. Kaplan–Meier survival curves between low- and high-TMB groups **(F)**. The Kaplan–Meier survival curves between the 4 groups **(G)**.

The immune-related functions are shown in a heatmap (Figure 8A); the inflammation-promoting, T cell co-inhibition, and immune checkpoint processes demonstrated a negative correlation in the HRG and a positive correlation in the LRG, whereas the difference in other immune cell types was small but not discrepant. The tumor mutation burden in HRG was higher than that in LRG, indicating a chemotherapy limitation for HRG (Figure 8B). The TIDE scores were higher in the LRG than in the HRG (Figure 8C); therefore, immunotherapy may not be appropriate for the LRG. Different somatic mutation changes were analyzed, and 15 highly mutated genes were selected. Mutations in TP53, TTN, MUC16, and CSMD3 are the most frequent in LUAD. Patients in the HRG presented with a higher TMB (94.51%) than those in the LRG (85.48%) (Figures 8D,E). Patients with a high TMB may benefit from 10 years of survival (*p* = 0.031). LRG patients with high TMB had the best OS, whereas HRG patients with low TMB had the worst 10-year OS (*p* < 0.001) (Figures 8F,G).

**FIGURE 9:**
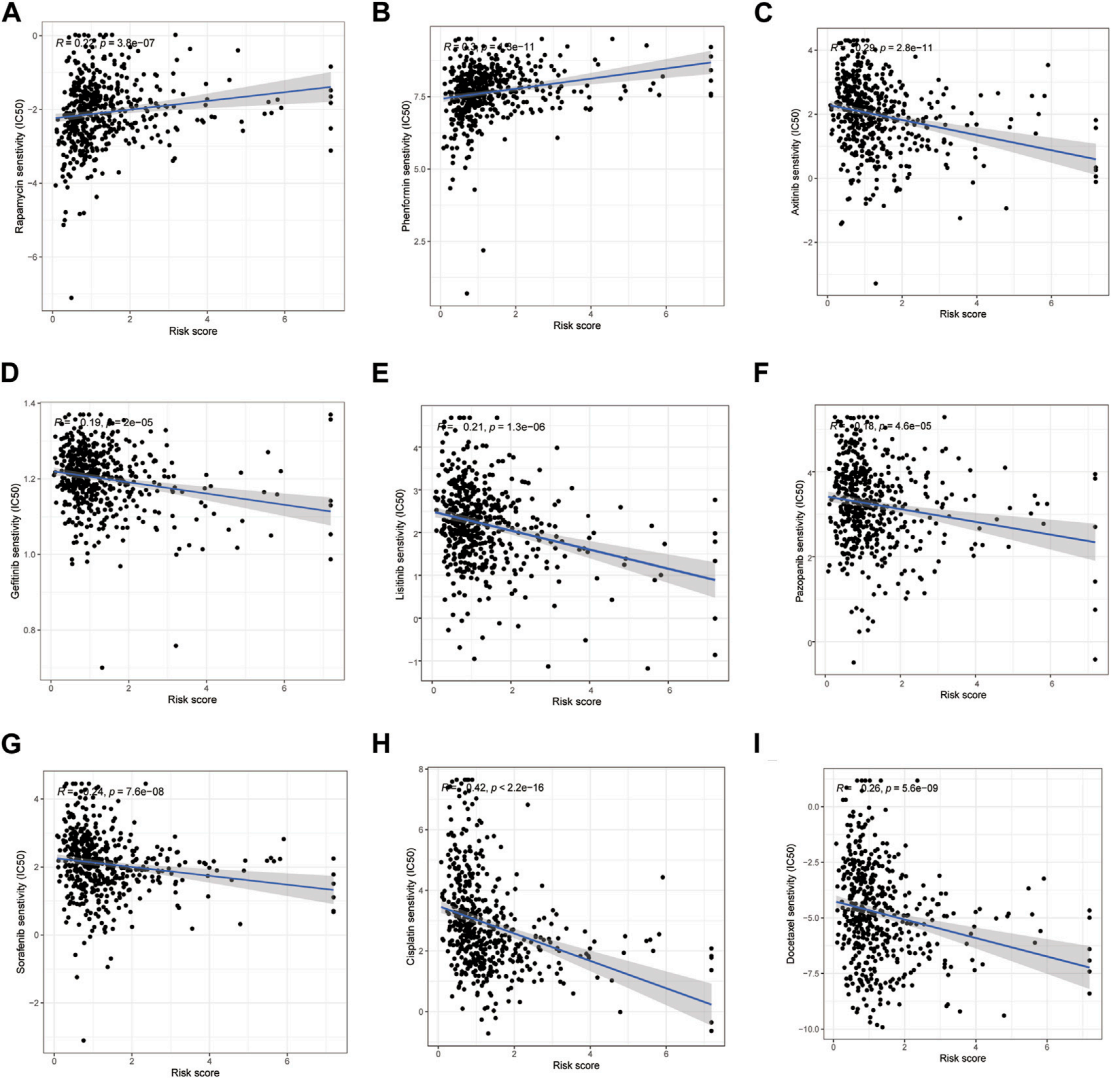
Correlation between the risk score and drug sensitivity. Drugs owned positive relation with the risk scores **(A, B)**. Drugs owned a negative relation with the risk scores **(C–I)**.

**FIGURE 10:**
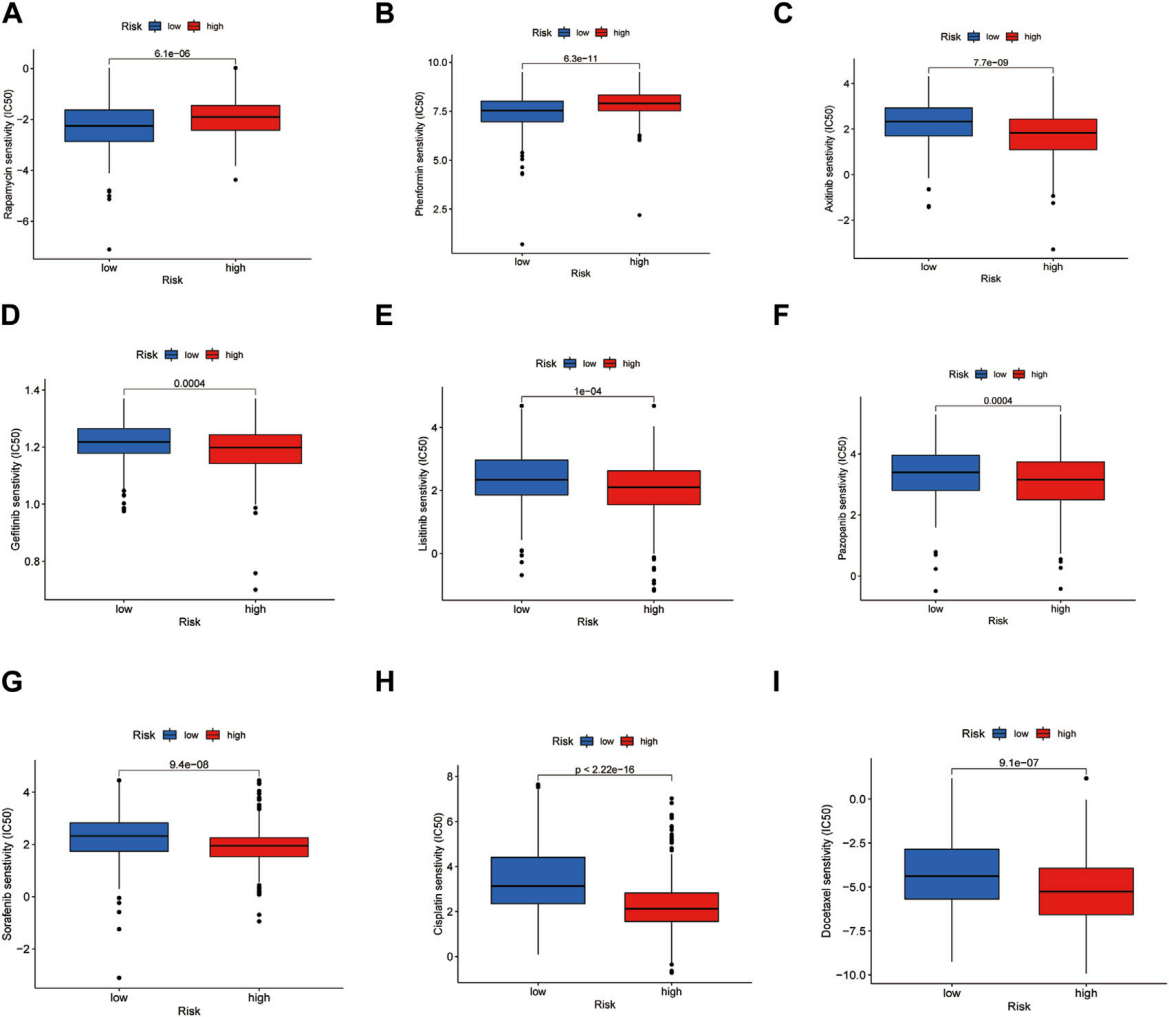
Drug sensitivity in LRG and HRG. Drugs were suitable for the HRG **(A, B)**. Drugs were suitable for the LRG **(C–I)**.

By comparing the drug sensitivities, differences were found in the half-limiting doses of LRG and HRG for several drugs. The sensitivities of the drugs in the two groups are shown in Figure 9. Among these drugs, rapamycin and phenformin showed a positive correlation with a higher risk score (Figures 9A,B) and greater sensitivity in the HRG (Figures 10A,B). However, targeted drugs such as axitinib, gefitinib, linsitinib, pazopanib, and sorafenib, and chemotherapeutic drugs such as cisplatin and docetaxel showed a negative correlation with higher risk scores (Figures 9C–I). These drugs were more effective in the LRG (Figures 10C–I).

### 3.9 Confirmation experiments

**FIGURE 11:**
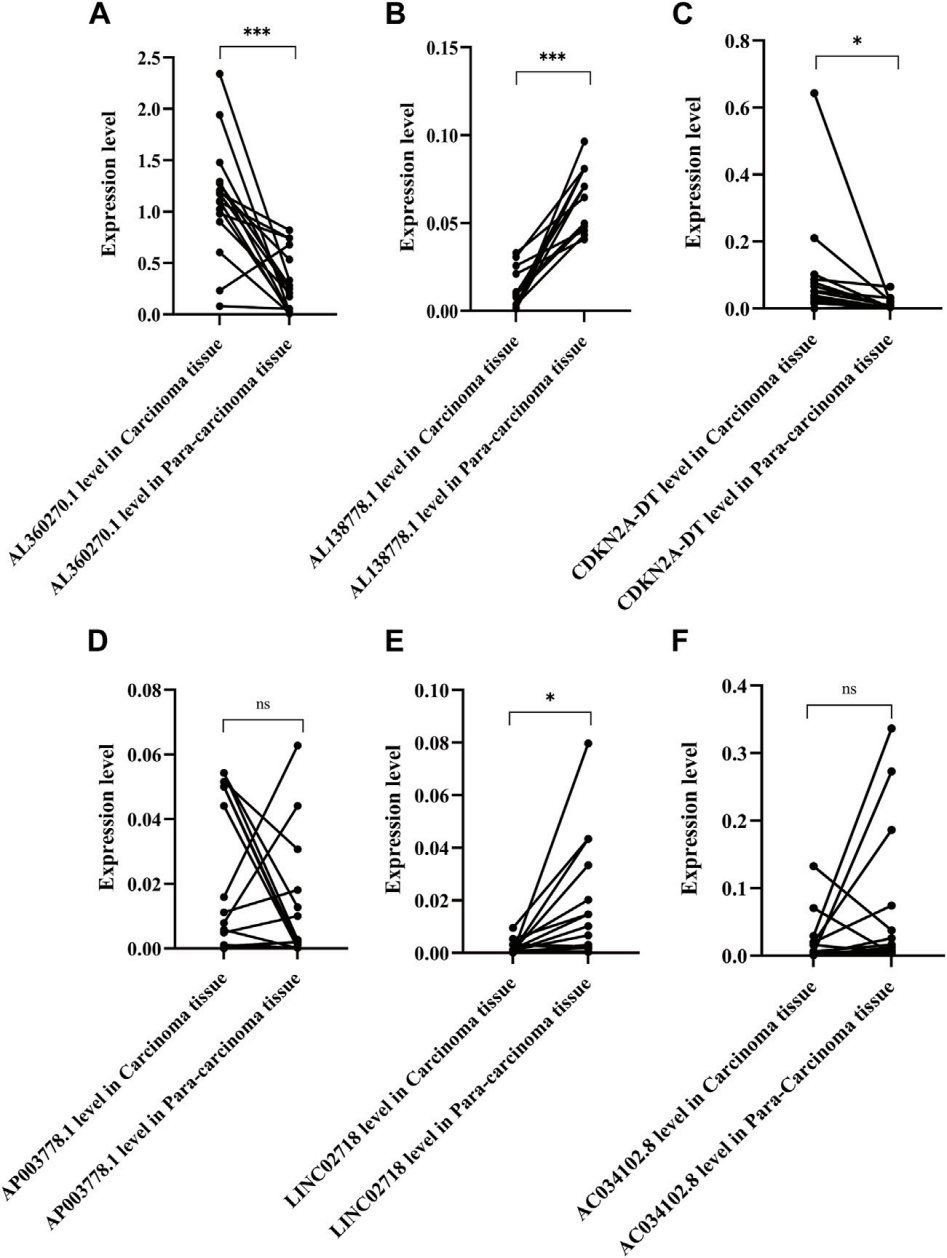
Expression level of the six lncRNAs of the model in 16 pair of adenocarcinoma and their para-carcinoma tissue. ***: **: *p* < 0.001, *p* < 0.01, ns: non-significant. **(A)**. Expression level of AL360270.1 in carcinoma and para-carcinoma tissue (C/P); **(B)**. Expression level of AL138778.1 in (C/P); **(C)**. Expression level of CDKN2A-DT in (C/P); **(D)**. Expression level of AP003778.1 in (C/P); **(E)**. Expression level of LINC02718 in (C/P); **(F)**. Expression level of AC034102.8 in (C/P).

We chose 16 pairs of adenocarcinomas and their para-carcinoma tissues to perform qPCR, which was tissue preservation solution. Basic and clinical characteristics of the 16 adenocarcinoma pairs were shown in Supplementary Table S1. The highest risk factor, AL360270.1, was expressed at higher levels in tumor tissues than in para-carcinoma tissues (*p* < 0.001) (Figure 11A). The protective factor AL138778.1 was significantly lower in tumor tissue than in para-carcinoma tissue (Figure 11B; *p* < 0.001). The expression of CDKN2A-DT was higher in tumor tissues than in normal tissues (Figure 11C; *p* < 0.05). The expression of LINC02718 was lower in tumor tissues than in para-carcinoma tissues (Figure 11E; *p* < 0.05). These four factors showed the same trend in differential expression as in the model. However, AP003778.1 and LINC02718 did not show any differential expression patterns in the sample test (Figures 11D,F).

According to the GO analysis, we found that the risk factors were closely related to cell movement and potential migration trends; therefore, we conducted a relative analysis of these six lncRNAs.

**FIGURE 12:**
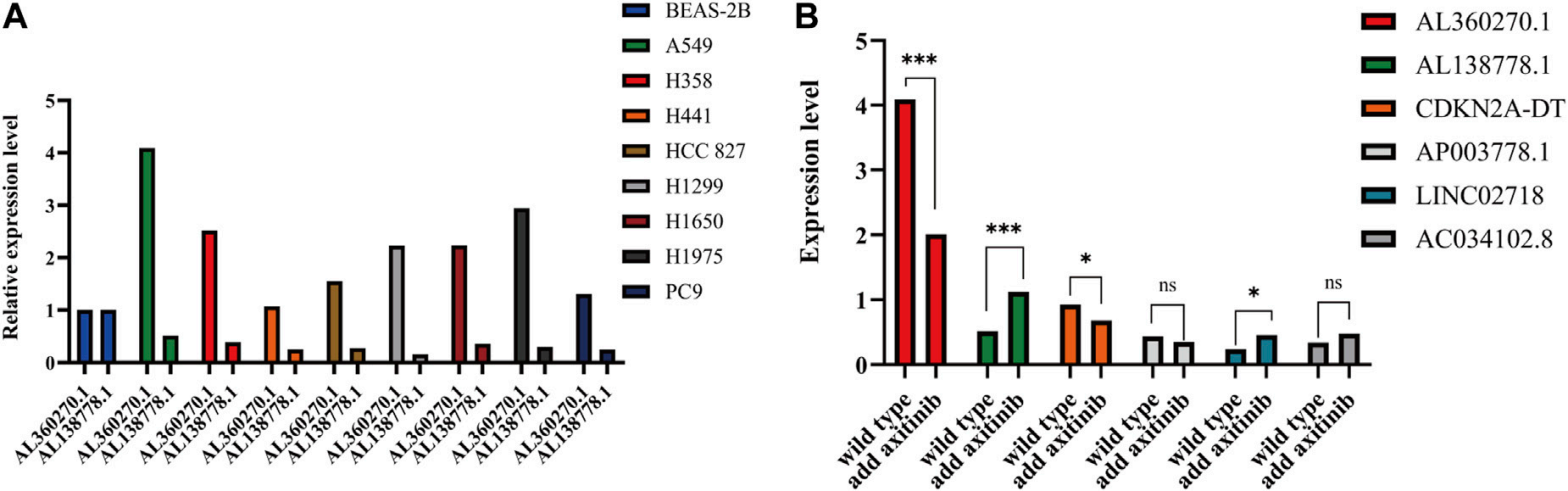
**(A)**. Expression level of AL138778.1 and AL360270.1 in 9 lung adenocarcinoma cell lines. **(B)**. In A549 cell line, the expression level of 6 lncRNAs grouped by wild and treated with axitinib.

AL138778.1 × (−0.998712907510809) and AL360270.1 × (0.672984389012466) were the most protective and malignant factors in our model. As for AL138778.1, a lower expression level was observed in tumor tissues than in para-carcinoma tissues. We selected nine LUAD cell lines, BEAS-2B, A549, PC9, HCC827, H1299, H1650, H1975, H358, and H441, to perform RT-qPCR and compare the expression levels of these two factors (Figure 12A). All the cell lines showed a higher expression level of AL360270.1 and a lower expression level of AL138778.1 than that of the BEAS-2B cell line.

To further confirm the selection of the potential target drug, we applied axitinib (10 nM) to wild-type A549 cells for 6 h in 24-well plates and then performed qPCR to determine the changes in the six lncRNAs. The results showed that AL360270.1, and AL138778.1, mutated significantly when compared to the wild type, which lowered the risk aspect. This trend was consistent with the model (Figure 12B).

The A549 cell line showed the highest expression levels (Figure 12A). Therefore, we chose to perform scratch wound and Transwell assays by knocking down AL138778.1, and AL360270.1. We constructed the AL138778.1 knock-down cell line that was compared with the AL138778.1 control cell line, and the AL360270.1 knock-down cell line that was compared with the AL360270.1 control cell line.

**FIGURE 13:**
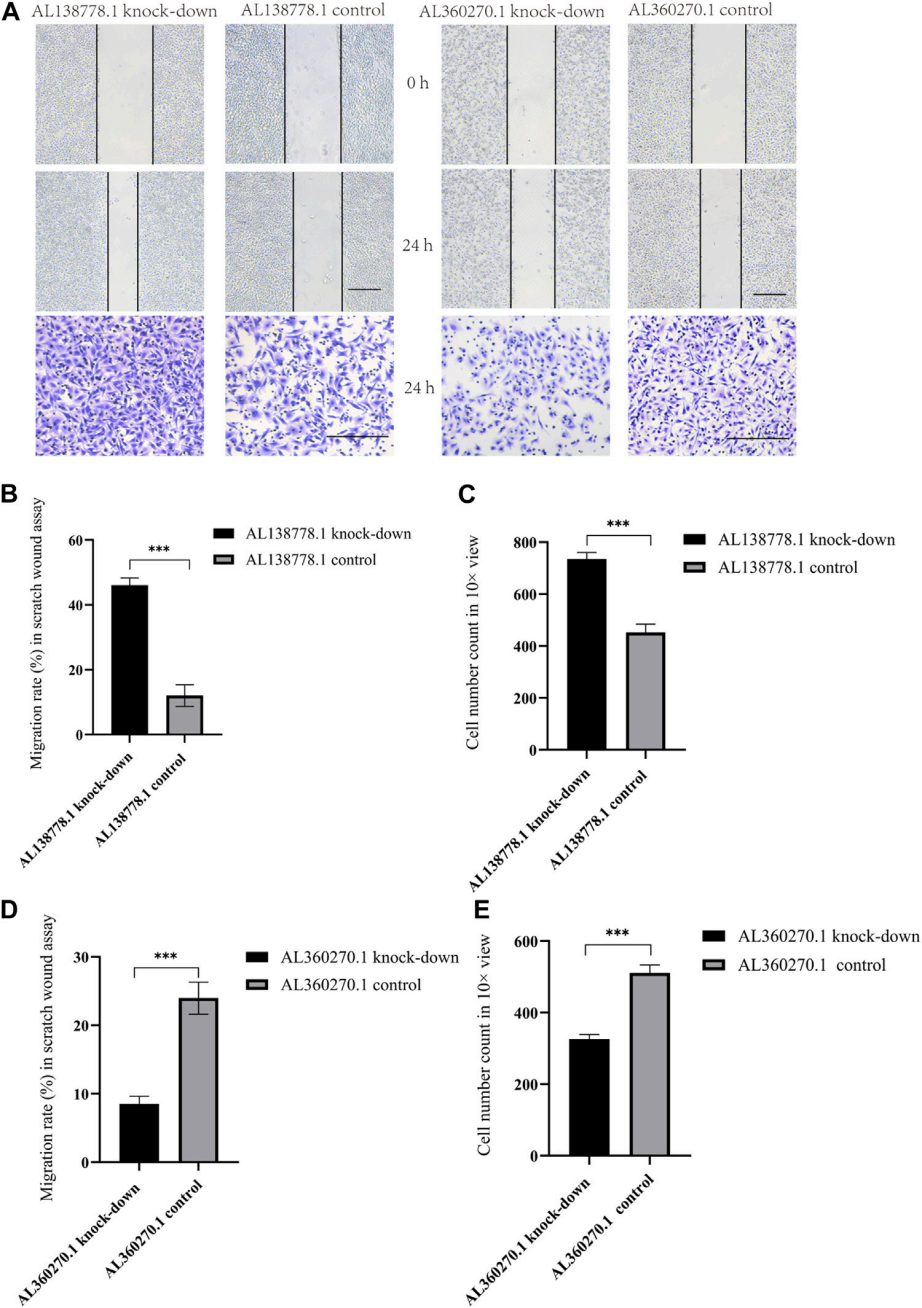
**(A)**. The major figure above were scratch wound assay and transwell in A549 cell line grouped by AL138778.1 knock-down vs. control, AL360270.1 knock-down vs. control. The transverse black line were the standard ruler of all figures. **(B)**. Migration rate (%) in scratch wound assay in AL138778.1 knock-down vs. control; **(C)**. Average Cell number count in 10× view in transwell for AL138778.1 knock-down vs. control; **(D)**. Migration rate (%) in scratch wound assay in AL360270.1 knock-down vs. control; **(E)**. Average cell number count in 10× view in transwell for AL360270.1 knock-down vs. control. ***: **: *p* < 0.001, *p* < 0.01, ns: non-significant.

After 24 h, the AL138778.1 knock-down cell line showed more migration ability than the control (Figure 13A). The relative migration rate = (migrated cell surface area/total surface area)×100. The relative migration rate was 46.08% vs. 12.75% (*p* < 0.001). A clear difference was observed (Figure 13B). Additionally, after 24 h, the AL360270.1 knock-down cell line showed less migration ability than the control (Figure 13A) The relative migration rate was 8.52% vs 23.98% (*p* < 0.001). A clear difference was observed (Figure 13D).

In the Transwell experiments, we found a significant difference between AL138778.1 knock-down and the control cell lines after 24 h of cultivation (Figure 13A). The AL138778.1 knockdown group showed a higher cell number on the Matrigel side than that of the control group (735 vs 452, *p* < 0.001) at ×10 magnification, indicating that AL138778.1 can protect the migration and metastasis of the tumor to some extent (Figure 13C).

In the Transwell experiments for AL360270.1 knock-down and its control set, a significant difference existed after 24 h of cultivation (Figure 13A). The AL360270.1 knock-down group showed fewer cells on the Matrigel side than that of the control group (326 vs 511, *p* < 0.001) at ×10 magnification. The results also indicated that AL360270.1 promotes tumor migration and metastasis (Figure 13E).

**FIGURE 14:**
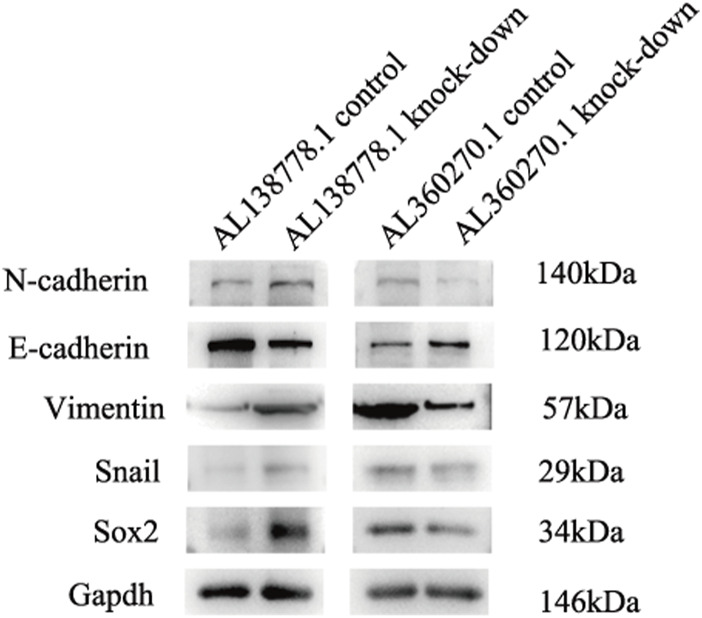
Western blot for migration features in A549 cell line grouped by AL138778.1 knock-down vs. control, AL360270.1 knock-down vs. control. The following items included N-cadherin, E-cadherin, Vimentin, Snail, Sox2, and proofed by Gapdh.

To further confirm this conclusion, we analyzed five protein factors related to CGs (Supplementary Figure S1). In the AL138778.1 knockdown group, there were higher expression levels of N-cadherin, Vimentin, Snail, and Sox2 and a lower expression level of E-cadherin than in the AL138778.1 control group (Figure 14). These results imply that the AL138778.1 knock-down cell line was more likely to metastasize than the AL138778.1 control cell line. In the AL360270.1 knock-down group, there was a lower expression level of N-cadherin, Vimentin, Snail, and Sox2, and a higher expression level of E-cadherin than in the AL360270.1 control group (Figure 14). These results indicated that the AL360270.1 knockdown group was less likely to metastasize than the AL360270.1 control group.

## 4 Discussion

A higher accumulation of Cu in cells can lead to severe consequences (Kim et al., 2008). Nevertheless, it has been reported that it is feasible to control normal intracellular Cu levels to selectively damage tumor cells (Masaldan et al., 2018). Several genes identified in this study, such as CDKN2A and ATP7A, have confirmed the feasibility of anti-tumor therapy in previous research. For example, the gene CDKN2A screened from TCGA, was shown to suppress tumor proliferation and influence cell cycle control (Rayess et al., 2012). The Cu transporter ATP7A is vital for the activation of lysyl oxidase (LOX) enzymes. Silencing ATP7A can inhibit LOX activity, which may trigger the loss of LOX-dependent metastatic mechanisms (Shanbhag et al., 2019).

However, intracellular Cu levels are related to metabolic and transportation mechanisms (Cobine and Brady, 2022). Studies have revealed an unconventional cell death mechanism most frequently influenced by protein lipoylation during the TCA cycle (Kahlson and Dixon, 2022). We found that cilium movement and microtubule bundle formation had the highest frequency in our model, which is consistent with the fact that microtubule clusters foster cell invasion in malignant tumors (Lupo et al., 2016). KEGG analysis showed that microRNAs, IL-17, and the p53 signaling pathway were the most abundant and enriched in cuproptosis-associated processes, which may potentially affect the oxidative stress even the prognosis in LUAD patients (Filaire et al., 2013). The involvement of the p53 signaling pathway in tumor suppression has been confirmed in various cell lines (Huang, 2021). As shown in the map tools of the two groups, TP53 missense mutations were the most frequent. Recent studies on immune cell infiltration and the tumor microenvironment have provided insights into immune cells. Researchers have demonstrated that higher levels of NK cells can suppress the proliferation of CD8+T cells and affect immune regulation (Sierra et al., 2021). The differentiation of T cells can affect the prognosis of patients with LUAD to some extent (Yu et al., 2020). Although the status of immune cells has outstanding value in anti-tumor therapy, the heat map of the expression and function of immune-related cells did not show a significant difference between the two groups. Patients in the HRG had lower TIDE scores than those in the LRG, indicating that immunotherapy may be limited to the HRG. Thus, in our model, patients in the LRG were more suitable candidates for targeted therapy. Consistent with our study, other studies have concluded that the clinical value of pazopanib could be more prominent in patients with higher risk factors (Xu et al., 2022). In addition, the IC50 drug susceptibility analysis suggested that patients in the HRG were more sensitive to phenformin, which can be used to target cancer cells and prevent relapse and metastasis (Krishnamurthy et al., 2014). Qin et al. have identified a novel prospective therapeutic target for cuproptosis (Qin et al., 2023). Similarly, our study used targeted drugs for LRG. Although patients with LRG exhibit a higher TIDE, which may lead to a poorer prognosis and less immunotherapy efficacy (Blumenschein, 2008), sorafenib, axitinib, pazopanib, and linsitinib may bring clinical benefits, which have been proven in previous trials (Leighl et al., 2017; Sun et al., 2018).

The lncRNAs affect the process of tumor metastasis by regulating the cell cuproptosis sensitivity such as CDKN2A, GLS and MTF1 (Xie et al., 2023). We demonstrated the predictive value of six CRs, including AL360270.1, AL138778.1, CDKN2A-DT, AP003778.1, LINC02718, and AC034102.8. Similarly, Liu et al. showed that the lncRNA AC034102.8 was a potential marker influencing the survival of patients with LUAD by pyroptosis (Liu et al., 2022). However, for these factors in our model, qPCR did not show differential expression in the carcinoma and para-carcinoma tissues. This may be due to AC034102.8 demonstrating a lower coefficient in the model. Although few studies have mentioned their function, AL138778.1 and AL360270.1 displayed their different capabilities for tumor migration and cell movement, which were in accordance with our model. Several studies have shown that CDK2N-DT promotes cancer (Tan et al., 2011). Similarly, our *in vitro* experiment also showed a high expression trend in carcinoma tissues when compared to that of para-carcinoma tissues. In addition, the transcription versions of AP003778.1, LINC02718, and AC034102.8 were long and mutable, so the lncRNA may vary among different versions. The function of these models may therefore change to some extent. This may be one of the reasons why the coefficient factor of these three lncRNAs (AP003778.1, LINC02718, and AC034102.8) was lower than that of the other three (AL360270.1, AL138778.1, and CDKN2A-DT).

Cu accumulation in cells can trigger tumor progression, and the GO analysis in our study highlighted microtubule-based movement, cilium movement, cilium organization, and cellular motility, which is different from the proposals based on previous studies that focused on the tricarboxylic acid cycle (Babak and Ahn, 2021). Although the relative expression of AL138778.1, AL360270.1, AP003778.1, and AC034102.8 has been reported in other studies, our study suggests that AL360270 is associated with the promotion of tumor metastasis and that AL138778.1 plays a role in the inhibition of metastasis. Scratch and Transwell experiments proved that the best and worst lncRNAs in our model were associated with cell movement and migration. Two of them have sufficient strength to balance the characteristics in our model, despite the potential influence of other lncRNAs. We chose N-cadherin, E-cadherin, Vimentin, Snail, and Sox2 to further confirm the feasibility of our model, because all these factors are closely related to the metastasis traits in LUAD (Han et al., 2022). Higher expression of N-cadherin, Vimentin, Snail, and Sox2 and lower expression of E-cadherin are always associated with tumor metastasis (Na et al., 2020). In addition, a correlation analysis of Cuproptosis genes with snail and Sox2 was conducted (Supplementary Figure S1). These Cuproptosis genes were closely related to AL138778.1 and AL360270.1 (Figure 1E). Therefore, we hypothesized that these five proteins may differ between AL138778.1 and AL360270.1 knock-down/control cell lines. After Western blotting, these factors were indeed found to be different in AL138778.1 and AL360270.1 knockdown/control cell lines, which further confirmed the migration features of our model.

However, this study had some limitations. Although we have validated the accuracy of our model, further experiments covering both *in vitro* and *in vivo* conditions are required. Second, larger public databases are needed to obtain more biological information to build up our evidence for this model. In future studies, these CRs should be validated in patients to test their target lncRNAs, and immunohistochemical analyses should be performed to determine the differences in related immune cells between the two groups.

## 5 Conclusion

We identified and validated six CR profiles (AL360270.1, AL138778.1, CDKN2A-DT, AP003778.1, LINC02718, and AC034102.8) by using various analytical methods and models. These CRs have clinical significance in predicting LUAD and may be used to evaluate the prognosis of patients with LUAD undergoing therapy.

## Data Availability

The original contributions presented in the study are included in the article/Supplementary Materials, further inquiries can be directed to the corresponding authors.
