# Supplementary material for: A cuproptosis-related lncRNA signature-based prognostic model featuring on metastasis and drug selection strategy for patients with lung adenocarcinoma
Source: Front Pharmacol. 2023 Sep 7;14:1236655. doi: 10.3389/fphar.2023.1236655 (PMC10513172; doi:10.3389/fphar.2023.1236655)
Supplement: Supplementary file 1 [file DataSheet1.docx]

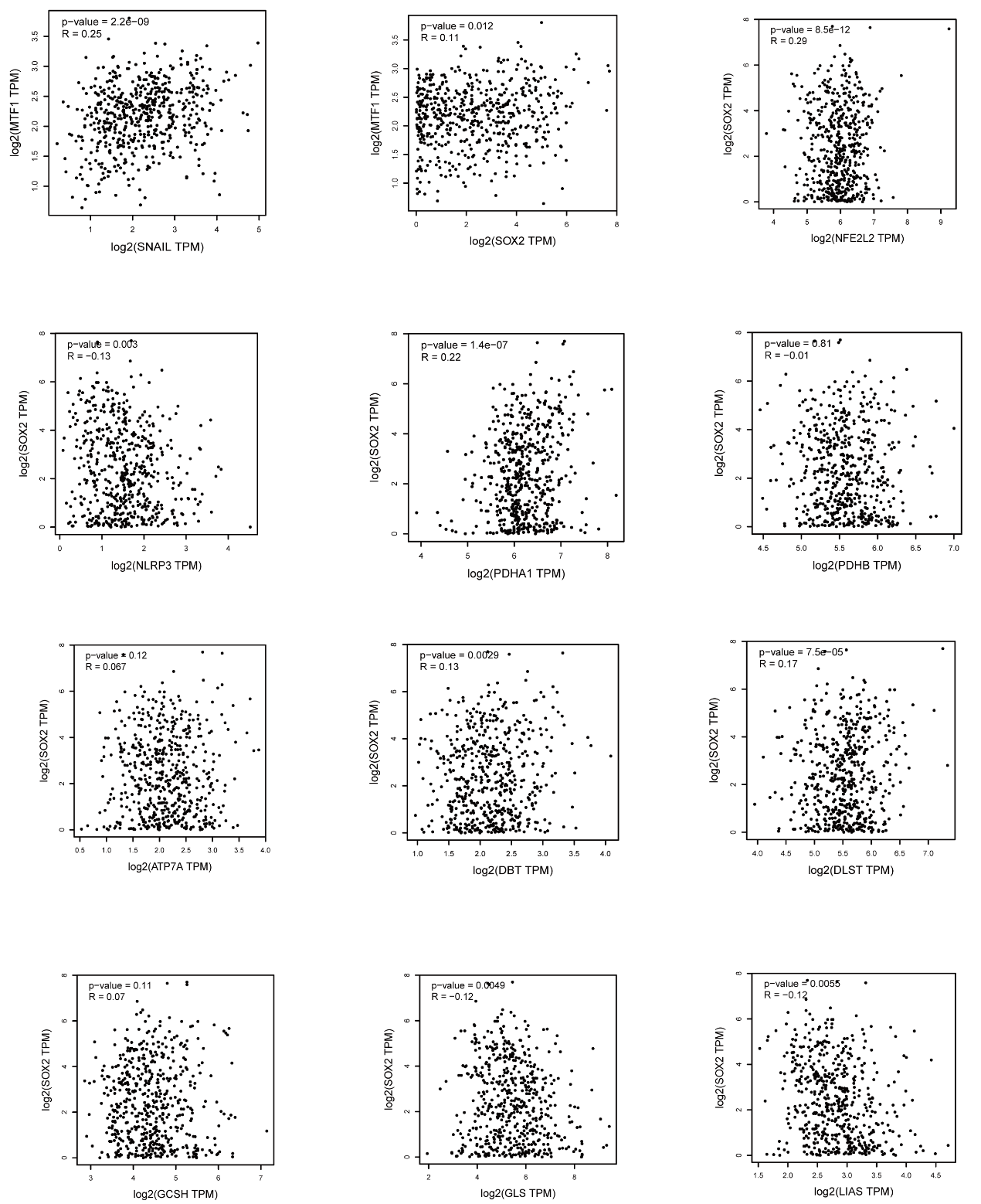


**Supplementary Figure 1.** Correlation analysis of Cuproptosis Genes with the Snail and Sox2. These Cuproptosis Genes were closely related with AL138778.1 and AL360270.1.


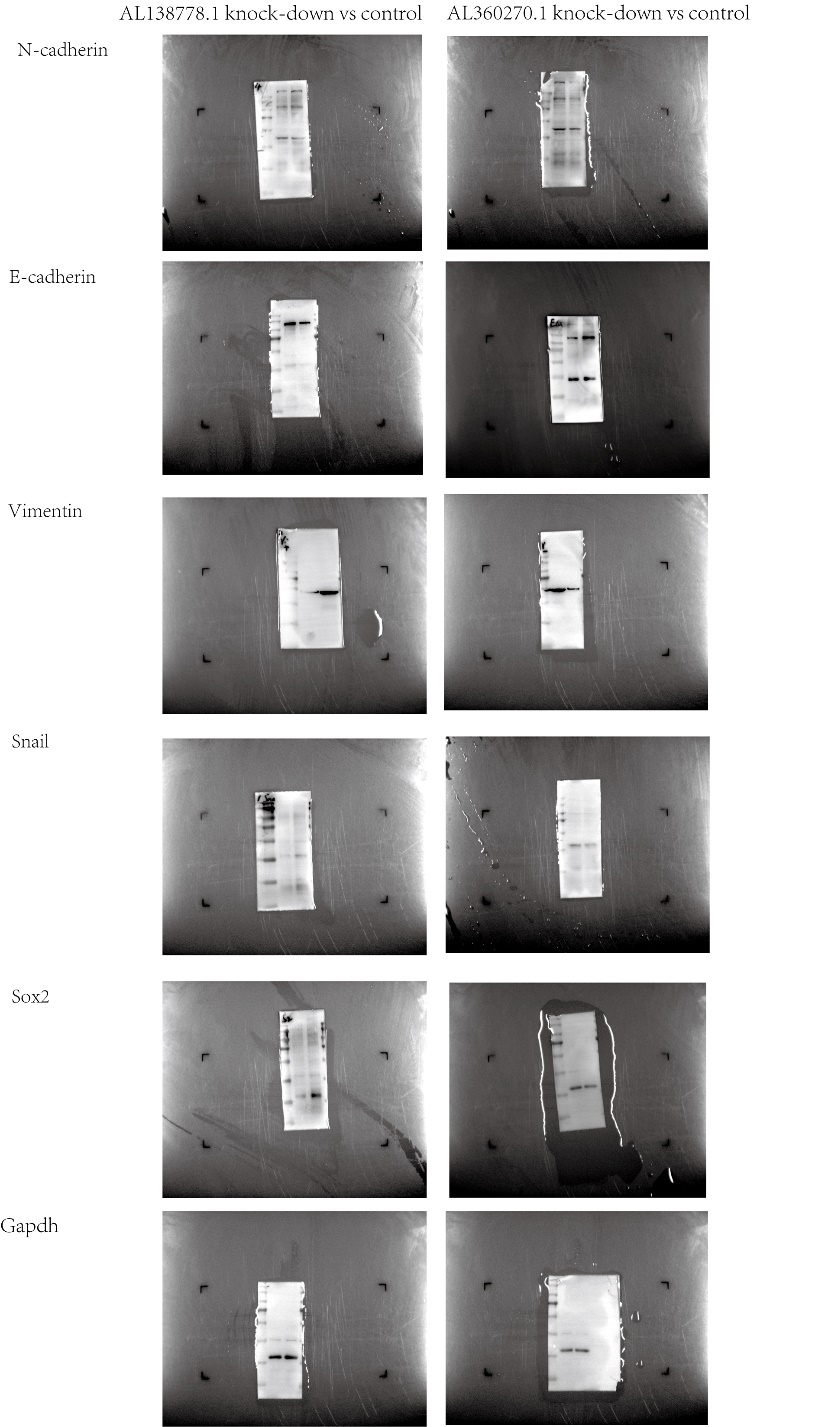


**Supplementary Figure 2.** The original gels of all the Western-blots included N-cadherin, E-cadherin, Vimentin, Snail, Sox2, and Gapdh.

**Supplementary Table 1.** The basic characteristic of the 16 pair of lung adenocarcinoma samples

| ID | Location | Pathological type | Pathological TNM stage |
| --- | --- | --- | --- |
| sample1 | left upper lobe | adenocarcinoma | T2N0M0 |
| sample2 | right upper lobe | adenocarcinoma | T1N0M0 |
| sample3 | left upper lobe | adenocarcinoma | T2N0M0 |
| sample4 | right upper lobe | adenocarcinoma | T3N2M0 |
| sample5 | right upper lobe | adenocarcinoma | T2N0M0 |
| sample6 | left lower lobe | adenocarcinoma | T2N1M0 |
| sample7 | left upper lobe | adenocarcinoma | T2N0M0 |
| sample8 | left lower lobe | adenocarcinoma | T2N2M0 |
| sample9 | left upper lobe | adenocarcinoma | T2N0M0 |
| sample10 | left lower lobe | adenocarcinoma | T2N2M0 |
| sample11 | left upper lobe | adenocarcinoma | T1N0M0 |
| sample12 | left upper lobe | adenocarcinoma | T2N0M0 |
| sample13 | right upper lobe | adenocarcinoma | T2N2M0 |
| sample14 | right upper lobe | adenocarcinoma | T3N2M0 |
| sample15 | right middle lobe | adenocarcinoma | T2N0M0 |
| sample16 | right upper lobe | adenocarcinoma | T2N0M0 |

**Supplementary Table 2.** Antibodies used in Western blots.

| Name |  | Number (No.) | Company |
| --- | --- | --- | --- |
| N-cadherin |  | CST13116s | Cell Signaling Technology |
| E-cadherin |  | AB1416 | Abcom |
| Vimentin  Snail |  | CST5741S  CST3879S | Cell Signaling Technology  Cell Signaling Technology |
| Sox2 |  | CST3579S | Cell Signaling Technology |
| Gapdh |  | RM2002 | Gene Tex |
